# Identification of MAGEC2/CT10 as a High Calcium-Inducible Gene in Triple-Negative Breast Cancer

**DOI:** 10.3389/fendo.2022.816598

**Published:** 2022-03-10

**Authors:** Heather K. Beasley, Sarrah E. Widatalla, Diva S. Whalen, Stephen D. Williams, Olga Y. Korolkova, Clementine Namba, Siddharth Pratap, Josiah Ochieng, Amos M. Sakwe

**Affiliations:** ^1^ Department of Biochemistry, Cancer Biology, Neuroscience and Pharmacology, School of Graduate Studies and Research, Meharry Medical College, Nashville, TN, United States; ^2^ Bioinformatics Core, School of Graduate Studies and Research, Meharry Medical College, Nashville, TN, United States

**Keywords:** calcium-sensing receptor, MAGEC2, TNBC, AP-1, breast cancer, cell proliferation, cell motility, calcium signaling

## Abstract

The expression of the melanoma/cancer-testis antigen MAGEC2/CT10 is restricted to germline cells, but like most cancer-testis antigens, it is frequently upregulated in advanced breast tumors and other malignant tumors. However, the physiological cues that trigger the expression of this gene during malignancy remain unknown. Given that malignant breast cancer is often associated with skeletal metastasis and co-morbidities such as cancer-induced hypercalcemia, we evaluated the effect of high Ca^2+^ on the calcium-sensing receptor (CaSR) and potential mechanisms underlying the survival of triple-negative breast cancer (TNBC) cells at high Ca^2+^. We show that chronic exposure of TNBC cells to high Ca^2+^ decreased the sensitivity of CaSR to Ca^2+^ but stimulated tumor cell growth and migration. Furthermore, high extracellular Ca^2+^ also stimulated the expression of early response genes such as FOS/FOSB and a unique set of genes associated with malignant tumors, including MAGEC2. We further show that the MAGEC2 proximal promoter is Ca^2+^ inducible and that FOS/FOSB binds to this promoter in a Ca^2+^- dependent manner. Finally, downregulation of MAGEC2 strongly inhibited the growth of TNBC cells *in vitro*. These data suggest for the first time that MAGEC2 is a high Ca^2+^ inducible gene and that aberrant expression of MAGEC2 in malignant TNBC tissues is at least in part mediated by an increase in circulating Ca^2+^ *via* the AP-1 transcription factor.

## Introduction

Triple-negative breast cancer (TNBC) remains the most aggressive and hard-to-treat breast cancer subtype due in part to lack of estrogen and progesterone receptors (ER, PR), as well as the human epidermal growth factor receptor 2 (HER2). Although the aggressiveness of the disease is more frequently diagnosed in younger and patients of African ancestry compared to Caucasian patients (1, 2), the underlying causes remain controversial. One of the most debilitating systemic cancer-associated changes that develop as breast and other solid cancers progress to more malignant and metastases-prone disease stages, is cancer-induced hypercalcemia (CIH). This often, overlooked abnormal increase in circulating Ca^2+^ is more likely to occur in at least 80% of patients with metastatic breast cancer due to the development of secondary disease lesions in the calcium-rich skeletal tissues (3–5). CIH may also develop in patients with rapidly growing tumors without evidence of bone metastasis due to increased secretion of parathyroid hormone-related protein (PTHrP) by the tumor cells (6, 7). In either case, the stimulation of osteoclast activity by tumor cell-derived PTHrP and other osteolytic factors leads to increased bone resorption and, eventually, increased circulating Ca^2+^ or CIH (8). Even though frequently diagnosed as mild or non-life-threatening, high circulating Ca^2+^ levels (>10.3 mg/dL) in breast cancer patients have been shown to be associated with aggressive tumors in premenopausal women (9) and larger tumors in post-menopausal women (10). While this suggests that hypercalcemia drives breast cancer progression, this notion and the underlying mechanisms remain poorly understood.

At the center of the Ca^2+^ sensing/signaling system is the ubiquitous cell surface Ca^2+^ sensor, the calcium-sensing receptor (CaSR), which is activated by slight increases in extracellular Ca^2+^ as well as other divalent cations and plays a central role in Ca^2+^ homeostasis (11, 12). Activation of the CaSR not only modulates the expression and/or activity of Ca^2+^ activated and Ca^2+^ binding proteins (13), but also the biosynthesis and secretion of osteolytic factors such as PTHrP (14). Like most stress conditions, an increase in circulating Ca^2+^ triggers the expression of early response genes such as c-FOS and EGR1 (15) which in turn may lead to the expression of genes involved in tumor progression. In addition to cell type-specific differences in the expression level of CaSR (16, 17), the activity of this receptor has also been shown to be modulated by specific mutations, including inactivating mutations in exon 7 of the receptor in breast cancer patients (18, 19). However, whether the expression levels, the activity of the CaSR, or both is sufficient to drive distinct proliferation and/or migration patterns of certain breast tumor cells at high Ca^2+^ remains unknown.

The melanoma associated antigen C2 (MAGEC2/CT10) is one of 60 identified cancer germline antigens that were previously described as cancer-testis antigens (20). MAGEC2, has been shown to be aberrantly expressed in squamous cell carcinomas, melanomas, sarcomas, myelomas, hepatocellular carcinomas, lung carcinoma, prostate adenocarcinoma, and breast carcinoma (20–22). Accumulating evidence suggests that MAGEC2 influences the progression and metastasis of breast cancer and other solid tumors (21–25) by mechanisms that include the promotion of epithelial-to-mesenchymal transition (23, 24), p53 stability (26) and stabilization of the activated form of STAT3 (27). Detection of MAGEC2/CT10 in patient tissues has also been shown to be an independent predictor of lymph node metastasis and recurrence of prostate cancer (22). To date, however, the physiological cues and the mechanisms underlying the increase in expression of this and related genes in malignant tissues remain unclear.

In this study, we sought to determine the effect of sustained high Ca^2+^ on the growth and motility of TNBC cells and to delineate the mechanism(s) underlying the increase in cell migration and proliferation at high extracellular Ca^2+^. We show that chronic exposure of TNBC cells to high extracellular Ca^2+^ led to a decrease in the sensitivity of the CaSR to Ca^2+^ but rather stimulated tumor cell growth and migration. Interestingly, high extracellular Ca^2+^ transiently induced the expression of early response genes that in turn induced the expression of genes including MAGEC2 that drive the growth and motility of the tumor cells. Overall, this study suggests that the aberrant expression of MAGEC2 and presumably related cancer-testis antigens in malignant solid tumors is triggered at least in part by the gradual increase in circulating Ca^2+^ that develops as breast cancer progresses to more malignant stages.

## Materials And Methods

### Cell Lines and Cell Culture

The breast epithelial cell lines 184A1 and MCF-10A, as well as the breast cancer cell lines MDA-MB-231, MDA-MB-468, BT-549, HCC38, HCC70, HCC1937, and HCC1806, were purchased from the American Tissue Type collection (East Rutherford, New Jersey, United States). The primary human breast epithelial cells (HMEC) were a gift from Dr. Jennifer Pietenpol (Vanderbilt Ingram Cancer Center). The cells were maintained in media and supplements recommended by the supplier and, except otherwise indicated, were supplemented with 10% fetal bovine serum (FBS, R & D Systems), 10 mM NaHCO_3,_ and Penicillin and Streptomycin, in a humidified incubator at 37°C, 5% CO_2_. The cells were passaged every 2-3 days.

### Microarray Experiments and Analyses

Total RNA was extracted from cells cultured in complete medium or complete medium supplemented with 3.0 or 5.0 mM Ca^2+^ for 48 h using the RNeasy kit (Qiagen, Valencia, CA). For microarray expression analysis, the quality of RNA samples was assessed using an Agilent Bioanalyzer. Target generation was performed by using the Ambion WT Sense reaction kit from Affymetrix and following the manufacturer’s protocol with 130 ng of intact RNA. The cDNA target was then enzymatically fragmented and end-labeled using the Affymetrix labeling reagents following manufacturer’s protocols. The cRNA, cDNA, and fragmented and end-labeled targets were assessed by Agilent bioanalysis to ensure that the amplified targets met the recommended smear range and to also assess whether fragmentation and end-labeling were complete. Only samples with a high RNA integrity number (RIN) number (>7) were used for hybridization. For Gene expression arrays, the requisite amount of target was added to the hybridization cocktail to give a final target concentration of ~25 ng/µl in the hybridization cocktail for a total of 2.5µg of fragmented targets hybridized to each array. The targets in hybridization cocktail were heat denatured, centrifuged, and then loaded on the human gene 2.0ST Affymetrix cartridge array and hybridized overnight in an Affymetrix Model 645 Hybridization Oven. After hybridization, the cartridge arrays were washed, and stained per standard Affymetrix protocols using an Affymetrix Fluidics Station 450. The arrays were then scanned in an Affymetrix 7G plus scanner and the resulting data were analyzed by Affymetrix Expression Console v.1.2 using an Robust Multi-array Average (RMA) normalization algorithm producing log base 2 results. Differential gene expression was assessed between replicate groups (n=4) using a moderated t-test and Benjamini-Hochberg multiple testing correction, with significance determined by adjusted p-value <0.05 and absolute value fold change >2.0. Hierarchical clustering was performed in GeneSpring on both averaged and non-averaged normalized expression values. Microarray datasets have been deposited and made available on the Gene Expression Omnibus (GEO) database with the accession number GSE189520 (https://www.ncbi.nlm.nih.gov/geo/query/acc.cgi?acc=GSE189520).

### Reverse Transcriptase and Real-Time PCR

For the validation of the identified genes, total RNA was isolated from selected breast cancer cells treated with complete medium supplemented with high Ca^2+^ (5.0 mM) for the indicated times. Control cells were those maintained in complete medium (~1.3 mM Ca^2+^) for the duration of the experiment. First-strand cDNA synthesis was performed using the iScript cDNA synthesis kit (BioRad) following the protocol provided by the manufacturer. This was followed by semiquantitative real-time PCR using individual TaqMan gene expression assays (**Table S1**) and TaqMan gene expression master mix (Life Technologies).

### Downregulation of MAGEC2/CT10 and FOS/FOSB in BT-549 Cells

Short hair-pin RNA (shRNA) directed against MAGEC2/CT10 in plasmid pGIPZ (Open Biosystems) were purchased from Thermofisher (Calsbad, CA). The plasmid DNA was purified using endofree maxiprep kits (Qiagen) and equal amounts (20 µg/10 cm dish) of the MAGEC2, c-FOS or FOSB and non-silencing scramble control (SCR) were used for transfection of BT-549 cells as previously described (28). Cells were sorted and/or selected with puromycin (2 µg/ml) for at least 10 days before use in experiments. The expression of MAGEC2/CT10, FOS, and FOSB was assessed by semi-quantitative RT-PCR and western blotting.

### Immunoblotting

Whole cell extracts were prepared in RIPA buffer (50 mM Tris-HCl, pH 7.4, 1% NP-40, 0.1% sodium deoxycholate, 150 mM NaCl, 1 mM EDTA and 1x protease inhibitor cocktail (Sigma) as previously described (29). Equal amounts of cleared lysates were separated in 4-12% NuPage gels (Invitrogen), transferred to nitrocellulose membranes, and probed with the indicated primary antibodies as previously described (29). Blots were revealed by enhanced chemiluminescence (Perkin Elmer). The expression of GAPDH or β-actin was used as an internal standard. The following antibodies were used: MAGEC2 (Abcam, Cambridge, MA), β-actin (Sigma-Aldrich, St. Louis, MO), Total ERK2, GAPDH, and CaSR (Santa Cruz Biotechnology Inc. Santa Cruz, CA), phospho-ERK1/2, c-Fos, and FosB (Cell Signaling Technology).

### Cell Proliferation and Migration Assays

Cells were plated into 24-well flat-bed plates at a density of 2 × 10^3^ cells per well in triplicate then incubated in medium containing high Ca^2+^ (5.0 mM) for up to 7 days with the culture medium changed every 3 days. At the end of each time point, PrestoBlue reagent (Invitrogen, Carlsbad, CA, USA) was diluted 1:10 in HBSS supplemented with 0.5 mM Ca^2+^ and 0.5 mM Mg^2+^ or the corresponding base medium for each cell line. The culture medium was aspirated and replaced with 100 µl of diluted reagent per well, followed by incubation at 37°C for up to 3 h. The proliferation and viability of the cells was determined by measuring the fluorescence according to the manufacturer’s instructions (Invitrogen). Cell migration was carried out in Boyden chambers as previously described (28). Migrated cells were fixed in 3.7% formaldehyde, then stained with crystal violet and counted.

### Genotyping of BC Cell Lines

Exponentially growing (60–80% confluency) breast cancer cells were harvested by scraping and were washed in ice-cold PBS. Cell pellets were either flash frozen at -80°C or used to isolate genomic DNA using the DNeasy blood and tissue DNA isolation kit (Qiagen). For determination of the CASR genotype, a 524 bp fragment containing the rs1801725, rs1042636, and rs1801726 loci in exon 7 of the receptor was amplified using Phusion High Fidelity PCR master mix (New England Biolabs) and the following primers: CASR-F CGAGGAGGTGCGTTGCAGCA and CaSR-R CCTCTGGCCGCTGGTCTCCA. Sequencing was performed using the ABI 3100 Genetic Analyzer according to the manufacturer’s protocol and read using sequence scanner version 2 (Applied Biosystems).

### Chromatin Immunoprecipitation

Cells were grown to 70-80% confluency, treated with or without high Ca^2+^ for 2, 4 or 48 h, and processed for ChIP assays using the ChIP Express IT kit according to the manufacturer’s instructions (Active Motif). Briefly, cells were fixed using 3.7% formaldehyde in PBS for 15 minutes at room temperature, then quenched with 100 mM glycine in PBS for 5 min and harvested by scrapping into ice-cold PBS. Cell lysates were enzymatically sheared using micrococcal nucleases, and the cleared lysates immunoprecipitated using antibodies against c-Fos or FosB (Cell Signaling Technology). After reversal of the cross-links, the associated DNA fragments were purified in spin columns and equal amounts used for semi-quantitative real-time PCR using primers targeting the MAGEC2 promoter (accession number: NM_016249.3).

### Luciferase Assays

The proximal MAGEC2 promoter including 90 bp downstream the transcription start site (-1008 - +90) was truncated by PCR and designated C2-P1 to C2-P5 as depicted in **Figure 6A**. Primers included a 5’ Hind III and 3’ Xho I restriction enzymes (Thermo Fisher Scientific). The truncated promoter segments were amplified from MCF-10A genomic DNA using Phusion High Fidelity PCR master mix (New England Biolabs), digested with the restriction enzymes, and cleaned using a DNeasy plasmid purification kit (Qiagen). The fragments were cloned into Hind III/Xho I digested and purified promoter-less pGL4 luciferase reporter vector (Promega). Cloning of the MAGEC2 promoter fragments was verified by restriction enzyme digestion and DNA sequencing. For luciferase assays, the MAGEC2 truncated promoter constructs and a control vector expressing Renilla luciferase were transfected into 293T cells in 6-well plates using Lipofectamine 3000 (Thermo Fisher Scientific, Waltham, MA, USA). Cells were cultured overnight at 37°C, media was changed the following day to complete medium with or without the indicated concentrations of Ca^2+^ and incubated for the indicated times. The cells were lysed using RIPA buffer, and luciferase activity was assessed by using the dual luciferase assay kit and analyzed following the manufacturer’s instructions (Promega). *Renilla* luciferase activity was used as the transfection control.

### Statistical Analysis

Data were analyzed using an unpaired *t*-test. If more than two groups were compared, one-way and two-way analysis of variance (ANOVA) were performed using GraphPad Prism (San Diego, CA, USA). For all statistical analyses p < 0.05 was considered statistically significant.

## Results

### Significance of CaSR Variants in the Growth and Sensitivity of Breast Cancer Cells to High Ca^2+^

**Figure 1:**
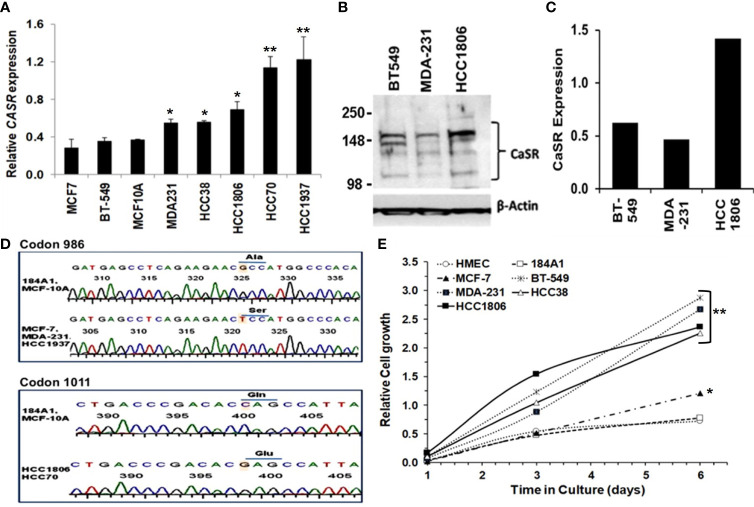
Significance of CaSR variants in the growth and sensitivity of breast cancer cells to high Ca^2+^. **(A)** Expression of CASR in breast epithelial and breast cancer cell lines was assessed by reverse transcriptase and real-time PCR (RT-PCR). **(B, C)** Basal expression of CaSR in the indicated breast cancer cells was assessed by western blotting **(B)** and quantified by using the NIH ImageJ software **(C)**. **(D)** Genotyping of exon 7 mutations in breast epithelial and breast cancer cells by DNA sequencing of a 524 bp fragment. Shown are chromatograms depicting changes of the nucleotide sequence at codons 986 (Ala to Ser) and 1011 (Gln to Glu). **(E)** Effect of high Ca^2+^ on the growth of breast epithelial and breast cancer cells *in vitro*. Cell proliferation/viability was assessed by using the PrestoBlue cell viability reagent. * denotes p < 0.05, ** denotes p < 0.01.

Most normal and malignant cell types that express the CaSR respond to extracellular Ca^2+^ as a first messenger to elicit cellular functions such as growth and motility (30, 31). The Ca^2+^ threshold for cell lines is quite distinct from that in living individuals due to the existence of an elaborate Ca^2+^ homeostatic system (32, 33). Consequently, high circulating ionized Ca^2+^ *in vivo*, is depicted as Ca^2+^ concentrations above the normal Ca^2+^ concentration of ~1.3 mM (4.4-5.2 mg/dL). In the case of cell lines and heterologous expression of the CaSR, Bai et al., showed that the effective concentration (EC_50_) of Ca^2+^ as an agonist for the CaSR overexpressed in HEK293 cells was ~4.1 mM (33). In the studies described herein, 3.0 and 5.0 mM Ca^2+^ concentrations were routinely used as high Ca^2+^ for TNBC cells. Thus using normal breast epithelial and a diverse set of breast cancer cell lines, we demonstrate that the expression of the CaSR at the mRNA level (**Figure 1A**) is cell type specific. We next confirmed this at the protein level in BT-549, MDA-MB-231 and HCC1806 TNBC cell lines (**Figures 1B, C**).

Given that polymorphisms at codons 986 (rs1801725) and 1011 (rs1801726) alter the sensitivity of the receptor to Ca^2+^ and are associated with high circulating Ca^2+^ (18, 19, 34), we sought to determine the genotype of the receptor at these loci in established breast epithelial and breast cancer cell lines. As depicted in **Figure 1D** and **Table 1**, normal breast epithelial cell lines such as MCF-10A and 184A1 express the wild type receptor at both codons, while breast cancer cell lines express the wild type (e.g. BT-549), the A986S (MCF-7, MDA-MB-231) or the Q1011E (HCC1806, HCC70) mutant receptors.

**Table 1:** Calcium-sensing receptor Exon 7 variants in normal breast epithelial and breast cancer cells.

Cell line	Source and Cat #	Disease	Race*	Exon 7 CASR Genotype**
184A1	ATCC-CRL-8798	Normal	White	AS
BT-549	ATCC-HTB-122	Ductal carcinoma	White	WT
MCF-7	ATCC-HTB-22	Adenocarcinoma	White	AS
MCF-10A	ATCC-CRL-10317	Normal	White	WT
MDA-MB-231	ATCC-HTB-26	Adenocarcinoma	White	AS
MDA-MB-436	ATCC-HTB-132	Adenocarcinoma	White	WT
MDA-MB-468	ATCC-HTB-130	Adenocarcinoma	Black	WT
HCC38	ATCC-CRL-2314	Ductal carcinoma	White	QE
HCC70	ATCC-CRL-2315	Ductal carcinoma	Black	QE
HCC1806	ATCC-CRL-2335	Acantholytic SCC	Black	QE
HCC1937	ATCC-CRL-2336	Ductal carcinoma	White	WT

*The Race of the patient from whom the biopsy was taken, ND, Not determined; ** Alleles at rs1801725 (codon 986) and at rs1801726 (codon 1011).

To determine whether the combination of the expression level and the genotype of the CaSR at the rs1801725 or rs1801726 locus influenced their proliferation at high Ca^2+^, we cultured the indicated breast cells lines in culture medium containing 5.0 mM Ca^2+^ for up to 6 days. Analysis of the cell viability revealed that most triple-negative breast cancer cells lines (MDA-231, BT-549, HCC1806, and HCC38) with wild-type or mutant CaSR (A986S or Q1011E) grew more efficiently at high Ca^2+^ than the luminal-A MCF-7 cell line which expresses low levels of the A986S mutant receptor. Likewise, immortalized normal breast epithelial cells or primary human mammary epithelial cells (184A1, HMEC) grew less efficiently at high Ca^2+^ (**Figure 1E**). To determine if the differences in the growth of these cells was associated with their response to transient increases in Ca^2+^, we compared the response of the A986S expressing MCF-7 and MDA-MB-231 cells to high Ca^2+^ by Ca^2+^ spectrofluorimetry. This analysis revealed that the amplitude of intracellular Ca^2+^ transients in response to high Ca^2+^ (3.0 mM) in MCF-7 cells was larger than that in MDA-MB-231 or MCF-10A cells (**Figure S1A**). Similarly, the amplitude of intracellular Ca^2+^ transients in the basal-like TNBC cell line HCC38 was larger than that of the HCC1806 (**Figure S1B**). We also assessed the viability/proliferation of the indicated breast epithelial and breast cancer cells at high Ca^2+^ and showed that the viability of MCF 7 cells was strongly impacted at high Ca^2+^ (**Figure S1C**). Based on these data, it is plausible to suggest that the decrease in the viability/proliferation of MCF-7 cells at high Ca^2+^ could be due to the more intense intracellular Ca^2+^ surge. However, these data also suggest that the expression level, the mutational status in exon 7 of the CaSR and the amplitude of intracellular Ca^2+^ transients in response to high extracellular Ca^2+^, is cell type specific.

### High Calcium Adaptation of Breast Cancer Cells Is Associated With Reduced Sensitivity of CaSR to Extracellular Ca^2+^ But Promotes Cell Growth and Motility

**Figure 2:**
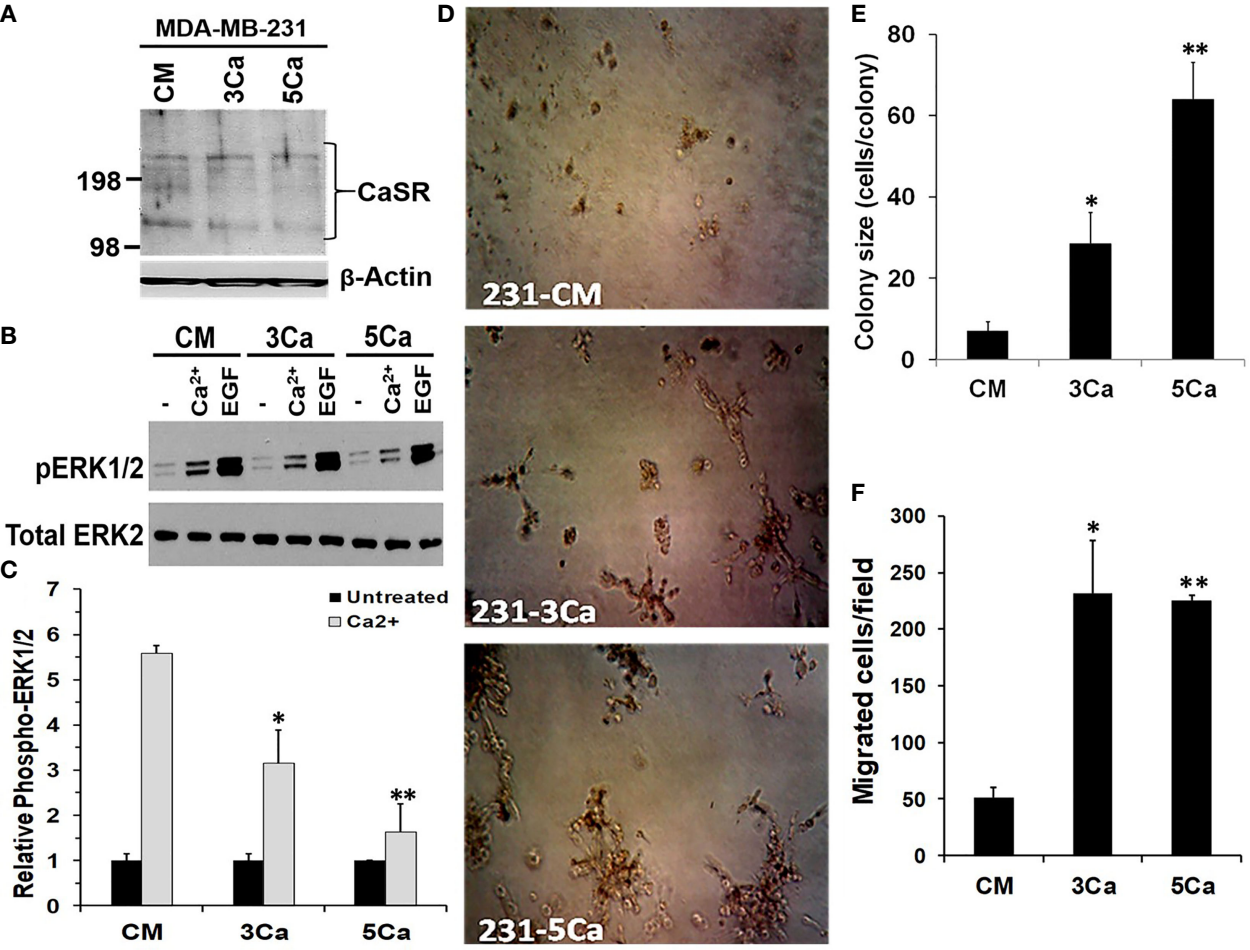
High calcium adaptation of breast cancer cells is associated with reduced sensitivity of CaSR to extracellular Ca^2+^ but promotes cell growth and motility. **(A)** MDA-MB-231 cells were cultured in complete medium supplemented with 3.0 or 5.0 mM Ca^2+^ for up to 6 weeks. The expression of CaSR in the Ca^2+^ adapted cells was assessed by western blotting; β-actin was used as the loading control. **(B)** The Control, 3.0 and 5.0 mM Ca^2+^ adapted MDA-MB-231 cells were treated with EGF (50 ng/ml) or high Ca^2+^ (5.0 mM) for 10 mins and the activation of ERK1/2 assessed by western blotting. **(C)** The protein bands representing phosphorylated ERK1/2 were quantified by using the NIH ImageJ. Bares represent active ERK1/2 relative to untreated control from independent experiments. **(D, E)** Control, 3.0 and 5.0 mM Ca^2+^ adapted MDA-MB-231 cells were seeded at 1000 cells/well in 96 well plates on growth factor reduced Matrigel and cultured in complete medium for up to 10 days. Cell colonies were captured microscopically using a digital camera **(D)** and colony sizes were assessed by manually counting the cells in each colony **(E)**. **(F)** Migration of Ca^2+^ adapted cells. Control, 3.0 and 5.0 mM Ca^2+^ adapted MDA-MB-231 cells in serum free medium were cultured for 24 h in Boyden chamber inserts and serum free medium containing 5.0 mM Ca^2+^ was used as the chemoattractant. Shown are the migrated cells/field from at least three independent fields. * denotes p < 0.05, ** denotes p < 0.01.

Up to 80% of advanced breast cancers are known to spread to Ca^2+^ rich skeletal tissues (4). To determine whether TNBC cells acquire tolerance to sustained high Ca^2+^, MDA-MB-231 cells were maintained in normal growth medium supplemented without or with 3.0 or 5.0 mM Ca^2+^ herein referred to as 3Ca and 5Ca cells respectively, for >6 passages over a six-week period (**Figures S2A, B**). Parental and the Ca^2+^ adapted cells were then tested for their ability to respond to high Ca^2+^ or EGF using the activation of MAP kinase ERK1/2 by western blotting as the read out (28, 35). **Figure 2A** reveals that the CaSR protein levels did not noticeably change following the continuous growth of MDA-MB-231 cells at high Ca^2+^. EGF-induced activation of ERK1/2 was not only robust, but also unaffected by the prolong culture of these cells at high Ca^2+^ (**Figure 2B**). However, transient (10 min) treatment of the parental cells with high Ca^2+^ (5.0 mM) led to a strong (5.5 fold) activation of ERK1/2, while a similar treatment of the 3.0 mM Ca^2+^- and 5.0 mM Ca^2+^-adapted cells led to a 3.5 fold and 1.7 fold change in the activation of ERK1/2 respectively, relative to control (**Figures 2B, C**). Collectively, these data demonstrate that while the CaSR protein levels remain unchanged, there is possibly desensitization of the CaSR to high Ca^2+^, leading to the downstream affects depicted by altered ERK activation.

We next assessed whether sustained high Ca^2+^ altered the ability of parental and high Ca^2+^ adapted MDA-MB-231 cells to form colonies in 3D cultures. Consistent with data in **Figure 1E**, the high Ca^2+^-adapted cells formed larger colonies compared to the parental cells (**Figure 2D**) and the extent of growth was Ca^2+^ concentration-dependent (**Figure 2E**). We also analyzed the ability of the high Ca^2+^ adapted cells to migrate in Boyden chambers by using serum free medium supplemented with 5.0 mM Ca^2+^ as the chemoattractant. **Figure 2F** and **Figure S2C**, show that compared to parental MDA-MB-231 cells, the 3.0 or 5.0 mM Ca^2+^-adapted cells showed a >4 fold ability to migrate in response to high Ca^2+^. Together, these data suggest that breast cancer cells adapt to high prevailing Ca^2+^ levels *via* reduced sensitivity of CaSR to the sustained high extracellular Ca^2+^, and that the high Ca^2+^ adapted cells are more efficient in migration and/or growth at high Ca^2+^. Together with data in **Figure 1**, these data support the notion that the physiological impact of high Ca^2+^ in various breast cancer cells may depend on the high Ca^2+^ modulated Ca^2+^ influx presumably *via* non-selective Ca^2+^ channels (36) and/or high Ca^2+^ modulated effectors downstream of the CaSR.

### Induction of Early Response and Malignancy Associated Genes in Breast Cancer Cells in Response to High Ca^2+^

**Figure 3:**
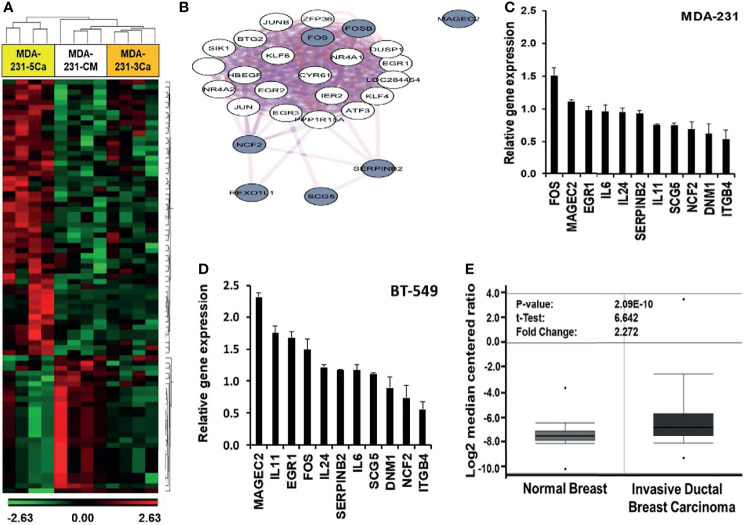
Induction of early response and malignancy associated genes as a response of breast cancer cells to sustained high Ca^2+^. **(A)** Heatmap of Gene expression profiling of high Ca2+ treated MDA-MB-231 cells. **(B)** Biological network relationship of up regulated (high Ca2+ inducible) genes (hashed nodes), pink edges are physical interactions and purple edges are co-expression associations (https://genemania.org/). **(C, D)** Validation of high Ca^2+^ inducible genes by RT-PCR in MDA-MB-231 **(C)** and BT-549 **(D)** TNBC cells. **(E)** Expression of MAGEC2 in normal breast (n = 61) and invasive ductal breast carcinoma (n = 396) was analyzed in the TCGA Breast dataset in oncomine.

Previous studies revealed that breast cancer cells respond to acute increases in extracellular Ca^2+^ by up regulation of early response genes such as FOS and EGR1 (15, 37). This prompted us to speculate that a decrease in the activity of the CaSR as depicted in **Figure 2** is not sufficient to explain the strong adaptive response of invasive breast cancer cells to sustained or breast cancer induced progressive increases in circulating Ca^2+^ in patients. To better understand the mechanism by which certain breast cancer cells acquire tolerance to high Ca^2+^, we performed genome-wide gene expression profiling using MDA-MB-231 cells. As shown in **Table 1**, MDA-MB-231 cells express the A986S variant of the CaSR. The MDA-MB-231 cells were cultured in complete medium supplemented without (CM, complete medium) or with either 3.0 or 5.0 mM Ca^2+^ for 48 hrs. Total RNA was extracted, hybridized on to Affymetrix Hu Gene 2.0 gene chips and the microarrays analyzed as described in materials and methods. From this analysis we observed that exposure of cells to 3.0 mM Ca^2+^ affected about 180 genes and that about 555 genes were affected by culturing the cells at 5.0 mM Ca^2+^ compared to the CM control. Of the differentially expressed genes in cells cultured at 5.0 mM Ca^2+^, at least 111 genes were up-regulated and 69 genes were downregulated at +/- 1.5 fold and p < 0.05 (**Figure 3A**).

In addition to the early response genes (FOS, FOSB), MAGEC2 (CT10), SERPINB2 (PAI-2), NCF2, SCG5, IL11, IL24 and IL6 were among the most up-regulated genes (**Table S2**) while DNM1 and ITGB4 were among the most down-regulated genes (**Table S3**). Analysis of pathways affected by the culture of TNBC cells at high Ca^2+^ revealed significant modulation of cancer related pathways such as senescence and autophagy, cytokine and inflammatory response, TGF-β signaling, energy metabolism, as well as regulation of epigenetic stress (**Table S4**).

To determine the known relationships of these genes in specific pathways, **Figure 3B** demonstrates that most of the high Ca^2+^ inducible genes (hashed nodes) have already been demonstrated as Ca^2+^ activated/dependent genes. Among these genes, the expression of SERPINB2 (PAI-2) has been demonstrated to be Ca^2+^-dependent (38, 39) and the expression of PAI-2 has been shown to correlate with prolonged overall and disease-free survival of breast cancer patients as well as sensitivity to treatment with tamoxifen (40–42). **Figure 3B** also shows that although MAGEC2 is known to be upregulated in malignant forms of many cancers (21–23, 25, 43), its function as a Ca^2+^ modulated protein has not been demonstrated. Analysis of interactions with other high Ca^2+^ inducible genes revealed that it interacts with the high Ca^2+^ inducible SSX1 (**Figure S3**), a member of the SSX family of transcriptional repressors.

The effect of high Ca^2+^ on the expression of these genes was validated by semi-quantitative RT-PCR using individual Taqman gene expression assays (**Table S1**) and total RNA isolated from MDA-MB-231 and BT-549 (**Figures 3C, D**), as well as from HCC1806 and MCF-7 (**Figure S4**) breast cancer cell lines. Consistent with the molecular heterogeneity of breast cancer, this analysis revealed that high Ca^2+^-induced expression of early response genes FOS/FOSB as well as genes associated with malignant tumors such as MAGEC2 was cell type specific. Interestingly, MCF-7 cells that express relatively low levels of the A986S mutant CaSR and are sensitive to high Ca^2+^, did not show significant upregulation of FOS or MAGEC2 (**Figure S4**). Meanwhile, the expression of MAGEC2 was strongly induced by high Ca^2+^ in BT-549 cells (**Figure 3C**) and only modestly in MDA-MB-231 cells (**Figure 3D**). To assess the clinical relevance of this finding, we show that MAGEC2 is significantly upregulated in invasive ductal carcinoma tissues compared to normal breast tissues according to TCGA Breast dataset in oncomine (**Figure 3E**). Interestingly, the expression status of MAGEC2 is strongly associated with the relapse-free survival of basal-like breast cancer patients and has little or no effect on the relapse-free survival of ER positive or ER negative breast cancer subtypes (**Figure S5**).

### MAGEC2 Is a High Ca^2+^ Inducible Gene in Certain Triple-Negative Breast Cancer Cells

**Figure 4:**
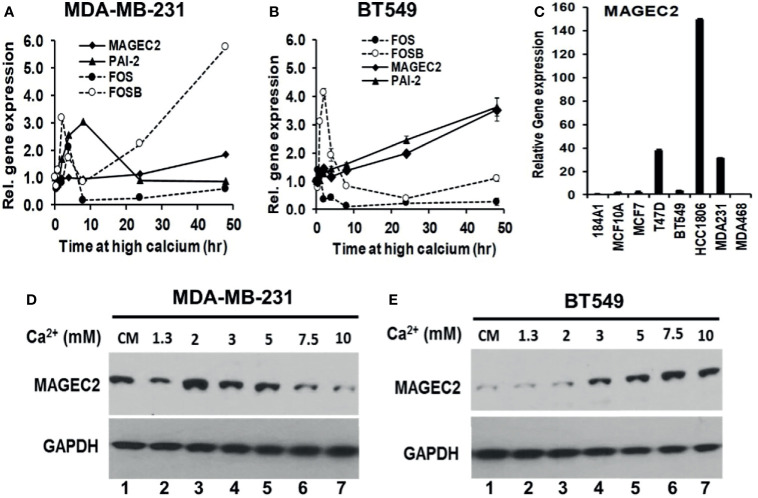
Induction of MAGEC2 expression by high extracellular calcium in TNBC cells. **(A, B)** Cells were cultured in complete medium with or without high Ca^2+^ (5.0 mM) for up to 48 h. Cells were harvested and total RNA extracted, and used for RT-PCR. Each point represents the expression of the indicated genes relative to GAPDH for MDA-MB-231 **(A)** and BT-549 **(B)** TNBC cells. **(C)** Total RNA was isolated from the indicated cell lines cultured in their respective complete media and the basal levels of MAGEC2 assessed by RT-PCR. **(D, E)** MDA-MB-231 **(D)** and BT-549 **(E)** TNBC cells were cultured in complete medium supplemented with the indicated concentrations of Ca^2+^, for 48 h. Cells were harvested and the expression of MAGEC2 protein was analyzed by western blotting.

Based on the microarray data, we speculated that the expression of early response FOS and/or FOSB may lead to the expression of cancer progression associated genes such as MAGEC2. To test this, we first determined whether the expression of these early response genes occurred at distinct time periods compared to that of malignancy associated genes. BT-549 and MDA-MB-231 cells were cultured in complete medium (containing 1.3 mM Ca^2+^) or complete medium supplemented with 5.0 mM Ca^2+^ following a time course for up to 48 h. The high Ca^2+^-induced expression of these genes was verified by semi-quantitative RT-PCR using individual TaqMan assays (**Table S1**, Life Technologies). As shown in **Figure 4A**, up-regulation of the early response genes FOS/FOSB at high Ca^2+^ occurred within 2 h in MDA-MB-231 cells followed by a modest increase in the expression of MAGEC2 and PAI-2. In BT-549 cells, however, there was a robust increase in the expression of FOSB within 2 h, followed by a steady increase in the expression levels of MAGEC2 and PAI-2 (**Figure 4B**).

To provide evidence for the difference in the response of MDA-MB-231 and BT-549 cells to high Ca^2+^ using MAGEC2 as the read out, we assessed the basal expression of MAGEC2 in these and other breast cell lines by RT-PCR. This analysis revealed that the basal expression of MAGEC2 in MDA-MB-231 cells was >10-fold that in BT-549 cells (**Figure 4C**). We next show that MDA-MB-231 cells indeed expressed higher levels of MAGEC2 at the protein level than BT-549 cells under basal culture conditions (**Figures 4D, E**, cf. lanes 1). Consistent with this difference in the basal expression of MAGEC2, we show that in MDA-MB-231 cells, the threshold for extracellular Ca^2+^ induced expression of MAGEC2 was ~2.0 mM and that higher concentrations of Ca^2+^ led to a consistent decrease in the expression of MAGEC2 (**Figure 4D**), and supports data in **Figure 4A**. On the other hand, treatment of BT-549 cells with various Ca^2+^ concentrations led to a consistent and Ca^2+^-dependent expression of MAGEC2 with a threshold at ~7.5 mM Ca^2+^ (**Figure 4E**), similar to data in **Figure 4B**. These data support the notion that distinct breast cancer cell lines not only have distinct thresholds but also varying potentials to adapt and to respond to the progressive increase in extracellular Ca^2+^.

**Figure 5:**
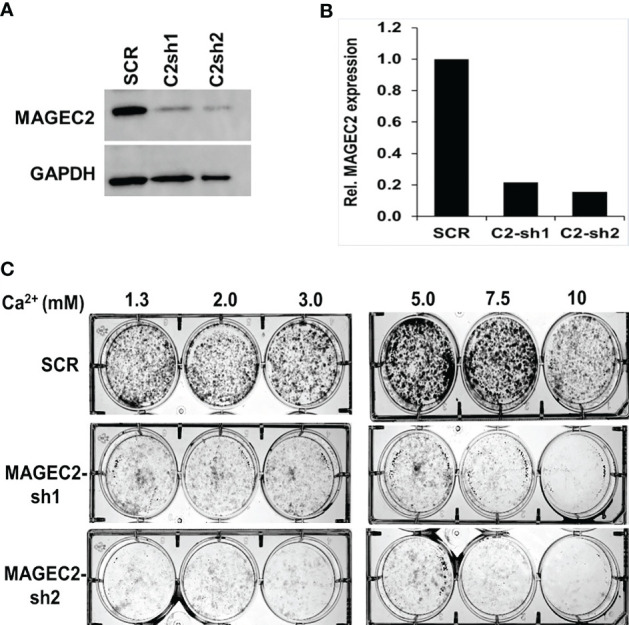
Effect of MAGEC2 down regulation on the growth of TNBC cells *in vitro*. **(A)** BT-549 cells were transfected with control (SCR) or shRNA plasmids (MAGEC2-sh1 and MAGEC2-sh2) targeting MAGEC2. The expression of MAGEC2 was confirmed by western blotting. **(B)** The protein bands were quantified using the NIH ImageJ software. **(C)** Control and MAGEC2 down regulated cells were cultured in complete medium supplemented with the indicated concentrations of Ca^2+^ and cultured for up to 10 days. Cells were fixed and stained with crystal violet and the images were digitally captured.

To confirm that the high Ca^2+^ inducible expression of MAGEC2 is required for cell growth, we transfected BT-549 cells with scramble control (SCR) or shRNAs targeting the coding sequence of MAGEC2 and verified the knockdowns by western blotting (**Figure 5A**). We then assessed the growth of the control (SCR) and two MAGEC2 shRNAs transfected cells at various concentrations of Ca^2+^ by clonogenic assays. Consistent with data in **Figures 4B, E**, we demonstrate that the growth of the control BT-549 cells was Ca^2+^ dependent with a threshold at ~7.5 mM Ca^2+^, while the growth of MAGEC2 shRNA transfected cells was inhibited and more so at Ca^2+^ concentrations >5.0 mM (**Figure 5B**).

### Induction of MAGEC2 Expression by High Ca^2+^ Is Mediated *via* the AP-1 Transcription Factor

**Figure 6:**
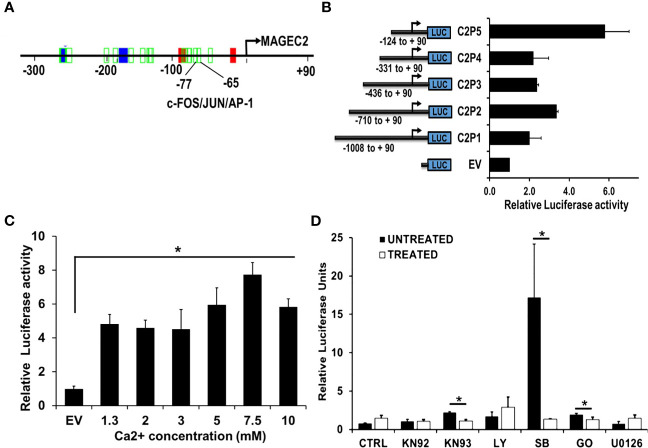
The proximal MAGEC2 promoter is activated by high Ca+. **(A)** The AP-1 transcription factor binding site on the proximal promoter of MAGEC2 was identified by using the Promo V3 software. B) The proximal promoter fragments of MAGEC2 truncated as indicated were cloned into pGL4 basic, then used to transfect HEK293T cells. For luciferase activity, HEK293T cells transfected with the empty vector (EV) or the MAGEC2 truncated promoter fragments and Renilla luciferase expressing vector (for transfection control) were cultured in complete medium with or without high (5.0 mM) Ca^2+^. Luciferase expression at high Ca^2+^ was assessed relative to the control. **(C)** HEK293T cells transfected with C2P5 from **(B)** above were cultured in complete medium supplemented with the indicated concentrations of Ca^2+^ and the luciferase activity assessed as in **(B)** above. **(D)** HEK293 T cells transfected as in **(B, C)** above were cultured in complete medium and high Ca^2+^ with or without the indicated inhibitors. Luciferase activity was measured 48 h post treatment as in **(B)** above. Luciferase activity as normalized to the DMSO control. EV: empty vector; DMSO: Dimethylsufoxide vehicle control; UNT (untreated, drug free); KN92: inactive Ca^2+^/camodulin kinase inhibitor; KN93 active Ca^2+^/camodulin kinase inhibitor; LY: LY 294002, PI3 kinase inhibitor; SB: SB 203580 p38 MAP kinase inhibitor; GO: Gö 6976, Ca^2+^ dependent PKC inhibitor; U0126, MEK inhibitor. * denotes p < 0.05, ** denotes p < 0.01, *** denoted p < 0.0001. Statistical significance was determined by Student’s T-Test and two-way ANOVA, where **(B, C)** were quantified to have a statistical significance of </= 0.05.

Given that the expression of FOS/FOSB early response genes at high Ca^2+^ peaked at ~2 h and steadily declined by ~8 hours (**Figures 4A, B**), we speculated that high Ca^2+^ induced expression of MAGEC2 may be mediated by these early response genes as components of the AP-1 transcription factor. To test this, we first show that the proximal promoter of MAGEC2 contains a bona fide AP-1/JUN/c-FOS binding site at -65 to -77 bp from the transcription start site (**Figure 6A**) based on the use of the Alggen prediction of transcription factor binding sites defined in the TRANSFAC database (Promo v3). To determine whether this promoter is responsive to high Ca^2+^, we carried out dual luciferase reporter assays using truncated versions of the MAGEC2 proximal promoter cloned upstream of firefly luciferase as depicted in **Figure 6B**. Analysis of luciferase expression revealed that the minimal proximal promoter denoted C2P5 (-124 to +90) containing the AP-1/JUN/c-FOS binding site was the most active at high Ca^2+^ (**Figure 6B**). We also show that the activity of the proximal promoter was Ca^2+^ concentration dependent with maximal activity at ~7.5 mM Ca2+ (**Figure 6C**) consistent with data in **Figures 4**, **5**

It is well established that the activation of c-Fos and its translocation to the nucleus is stimulated by its phosphorylation (44–48). We speculated that certain Ca^2+^ activated protein kinases may be involved in driving the expression of MAGEC2 at high Ca^2+^. For this study, we used Ca^2+^/calmodulin kinase inhibitor (KN-93 and KN-92 as the respective control), p38 MAP kinase inhibitor (SB203580), a MEK1/2 inhibitor (U0126), a phosphatidylinositol 3-Kinase (PI3K)inhibitor (LY294002), and a conventional protein kinase C isoform inhibitor (Gö6983). To test this, we treated HEK293T cells transfected with the minimal proximal MAGEC2 promoter (C2P5) in medium supplemented with high Ca^2+^ with or without the indicated kinase inhibitors for 48 hours, and assessed the luciferase activity. This analysis revealed that inhibition of Ca^2+^/calmodulin kinase, p38 MAP kinase, and Ca^2+^-dependent protein kinase C isoform blocked the Ca^2+^ induced luciferase activity (**Figure 6D**). This suggests that these kinases are, in part, responsible for the activation and subsequent transcriptional activity of c-FOS at high Ca^2+^.

### High Ca^2+^ Inducible Expression of MAGEC2 Is Mediated by c-FOS/AP-1 Transcription Factor

**Figure 7:**
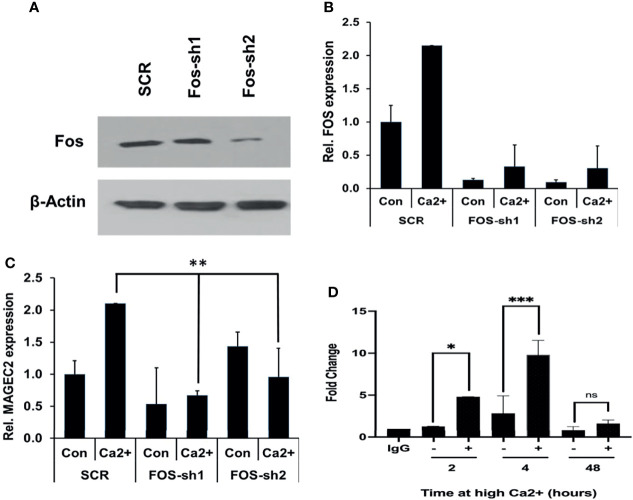
High Ca^2+-^induced expression of MAGEC2 is attenuated following downregulation of cFOS. **(A)** BT-549 cells were transfected with control (SCR) or shRNA plasmids (FOS-sh1 and FOS-sh2) targeting c-FOS. The expression of c-FOS was confirmed by western blotting. **(B, C)** BT-549 cells transfected with control or c-FOS targeting shRNAs were cultured in complete medium without (Con) or with high Ca^2+^ (Ca^2+^) for 48 h. The cells were harvested and the expression of c-FOS **(C)** and MAGEC2 (D) assessed by RT-PCR. **(D)** Parental BT-549 cells were grown to 70-80% confluency, then treated with or without high Ca2+ for the indicated times. Cells were subsequently cross-linked and processed for ChIP assays as described in materials and methods using antibodies against c-FOS. Purified DNA fragments were analyzed by real-time PCR and presented as fold change relative to IgG control. Con, complete medium control; Ca^2+^, complete medium supplemented with high Ca^2+^. * denotes p < 0.05, ** denotes p < 0.001, *** denotes p < 0.0001, ns denotes not significant.

As critical components of the AP-1 transcription factor, c-FOS and its homolog FOSB detected in our microarray data may contribute to the expression of MAGEC2. We first assessed whether downregulation of either FOS or FOSB could affect the transcription of MAGEC2 at high Ca^2+^. Here, we show that downregulation of c-FOS protein (**Figure 7A**) or mRNA (**Figure 7B**) in BT-549 cells was associated with a significant decrease in the transcription of MAGEC2 at high Ca^2+^ (**Figure 7C**).

Finally, we tested whether c-Fos bound to the promoter of MAGEC2 by chromatin immunoprecipitation. In this assay, antibodies to c-Fos were used for the immunoprecipitation, and primers for the minimal proximal promoter (C2P5) were used to amplify the recovered DNA fragments from BT-549 cells cultured in complete medium supplemented with high Ca^2+^ for 2, 4, and 48 hours. This analysis revealed that high Ca^2+^ enhanced the binding of c-Fos to the MAGEC2 promoter in a time dependent manner with maximal binding at ~4 h (**Figure 7D**). Together, these data suggest that MAGEC2 is a Ca^2+^-inducible gene in TNBC cells and that the aberrant expression of this gene in malignant tissues is at least in part, mediated *via* c-Fos and the AP-1 transcription factor.

## Discussion

Dysregulation of systemic Ca^2+^ homeostasis, especially in patients with high-grade tumors, is common, due to increased secretion of PTHrP and the development of co-morbidities such as cancer-induced hypercalcemia (CIH) (7, 49–53). High extracellular Ca^2+^ is known to promote the growth and motility of breast cancer cells *via* the CaSR (54–56), but the mechanisms remain not only complicated, but also poorly understood. This is especially true for the molecularly heterogeneous breast cancer in which circulating Ca^2+^ is associated with larger and aggressive tumors in post-menopausal women and pre-menopausal women, respectively (9, 10). In this study, we sought to determine the effect of sustained high Ca^2+^ on the growth and motility of TNBC cells and to delineate the mechanism underlying the increase in cell growth and motility at high extracellular Ca^2+^. We show that sustained high extracellular Ca^2+^ decreased the sensitivity of CaSR to Ca^2+^ but rather stimulated tumor cell growth and migration. Our data also reveal that the expression levels, mutational status in exon 7 of the CASR as well as the Ca^2+^ influx dynamics do not completely explain the distinct responses of breast epithelial and breast cancer cell lines to an increase in extracellular Ca^2+^. However, high extracellular Ca^2+^ provokes the expression of early response genes that in turn lead to the expression of malignancy associated genes that presumably drive the growth and motility of the tumor cells. The identification of MAGEC2 as a Ca^2+^ inducible gene supports this novel paradigm in Ca^2+^sensing and signaling in TNBC cells. Overall, this study suggests for the first time that the aberrant expression of MAGEC2 that is frequently observed in malignant solid tumors may at least in part be mediated by a cancer-induced increase in circulating Ca^2+^.

Although the CaSR plays a critical role in systemic Ca^2+^ homeostasis and promotes tumor cell growth and migration (54–56), the underlying mechanisms remain poorly understood. Previously, our lab showed that expression of CASR variants at rs1801725 is associated with a higher risk of developing secondary neoplastic lesions in the lungs and bone (19). We genotyped several breast cancer cell lines to determine if they have the wild-type CASR or the mutated CASR (**Table 1**). We show that, along with the heterogeneity among breast cancer cell lines, there are also differences among the cell lines as to how the cells respond to high Ca^2+^ *in vitro*. As predominantly inactivating mutations, CASR variants at rs1801725 and rs1801726 may contribute to the desensitization of the receptor to Ca^2+^ (57–61) and as such, may shift the response of tumor cells to higher set points of Ca^2+^. This reduced sensitivity of the CaSR may not only blunt the response of tumor cells to circulating Ca^2+^ but also facilitate the growth and motility of tumor cell *via* cytosolic Ca^2+^ mediated processes. Interestingly, previous studies reported that expression of the wild type and the activating R990G CaSR mutant in exon 7 of the receptor were both necessary and sufficient to induce humoral hypercalcemia of malignancy in lung squamous cell carcinoma xenograft tumor bearing mice (62). However, whether the expression of exon 7 inactivating CASR mutants in TNBC cells also influence the development of hypercalcemia remains to be fully elucidated.

In the tumor microenvironment and especially at metastatic sites, Ca^2+^ is a predominant but one of the least studied factors in TNBC biology. Although it is well established that circulating Ca^2+^ is associated with cancer progression (9, 10), the effects of high Ca^2+^ on tumor growth varies from one cancer type to another (54, 63, 64). For instance, high extracellular Ca^2+^ inhibits the proliferation of colon cancer and parathyroid cells (54, 65) but stimulates both the proliferation and metastatic potentials of breast and prostate cancer cells (55). Our study reveals that for the same cancer type, the response of various cancer cells to high Ca^2+^ is cell type specific. Whether this depends on the expression level and/or the expression of activating or inactivating mutant CaSRs by the cells is still poorly understood. This is further complicated by the diverse and often cell type dependent Ca^2+^ entry mechanisms (65, 66), the several Ca^2+^ dependent factors (15, 67, 68), signaling pathways (11, 13, 17, 69, 70) and a plethora of cellular functions modulated by an increase in cytosolic Ca^2+^ (69, 70). This notwithstanding, as cancer progresses to the more malignant stages, patients inevitably develop cancer-induced hypercalcemia (49–52) which may invariably influence the progression of the disease *via* the CaSR or independent of CaSR.

**Figure 8:**
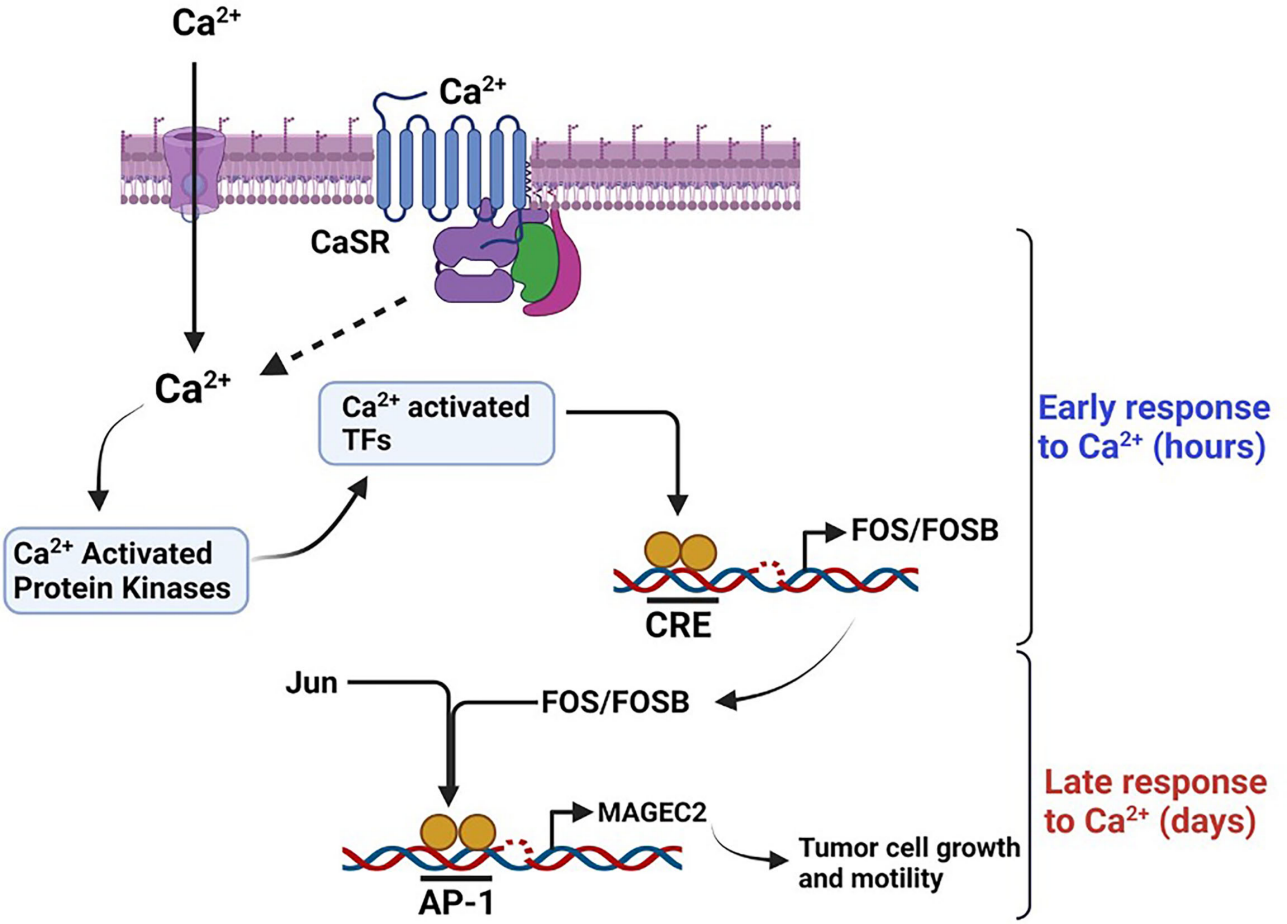
Schematic diagram depicting the effect of high Ca^2+^ on the expression of MAGEC2 in TNBC cells. High extracellular Ca^2+^ acting *via* the CaSR provokes store operated Ca^2+^ entry, the subsequent surge in intracellular Ca^2+^activates Ca^2+^ activated kinase such as conventional PKC isoforms which in turn activate immediate early Ca^2+^ activated transcription factors such as CREB/ATF4. These transcription factors lead to the expression of early response genes such as c-FOS which upon dimerization with Jun proteins bind to the AP-1 site on target genes such as MAGEC2 and provoke their transcription. Up regulation of MAGEC2 may then facilitate the adaptation of the cells to high Ca^2+^ and enhance their growth and metastatic properties.

Based on data from this study, it is possible that the outcome of breast cancer may be dictated by the response of tumor cells to progressive increase in Ca^2+^ as the disease progresses. Our data suggest that high Ca^2+^ adapted cells may develop into larger more aggressive tumors (9, 10), while Ca^2+^-sensitive tumor cells may develop into slow growing, less aggressive tumors. As illustrated in **Figure 8**, we propose the novel paradigm that an increase in cytosolic Ca^2+^ activates Ca^2+^ responsive protein kinases that activate Ca^2+^ responsive transcription factors (71, 72) and that in turn lead to the expression of early response genes (ERGs) such as c-FOS (15, 44–46, 73, 74). Dimerization of c-Fos with Jun proteins to form active AP-1 transcription factors (44, 47, 74), potentially drives the high Ca^2+^ inducible expression of malignancy-associated genes such as MAGEC2. Based on this model, it is possible that high extracellular Ca^2+^ exerts a selection pressure to enable tumor cells to either become less sensitive to Ca^2+^ but more aggressive or more sensitive to Ca^2+^ and less aggressive. This is supported by previous studies showing that high circulating Ca^2+^ is associated with aggressive tumors in premenopausal women and larger tumors in post-menopausal women (9, 10). Consistent with this notion, it is also possible that TNBC cells with the propensity to spread to Ca^2+^-rich skeletal sites may be those that become insensitive to high Ca^2+^ or are high Ca^2+^ adapted following a priming stage dictated by a progressive increase in circulating Ca^2+^ as the disease progresses. It is possible that the survival of Ca^2+^ adapted cells may be sustained by high Ca^2+^ inducible expression of genes that influence tumor cell growth and/or motility such as MAGEC2.

Up regulation of MAGEC2 and other high Ca^2+^ inducible genes that influence the growth and motility of tumor cells may be invaluable in the adaptation of tumor cells to high Ca^2+^ as depletion of this gene strongly attenuated the growth of the cells in either normal or high Ca^2+^ conditions. High Ca^2+^ and especially if sustained for a relatively long period may be stressful to cells that are not adapted to higher than normal circulating levels. Without adaptive mechanisms, including modulation of various Ca^2+^ channel activity, sustained high Ca^2+^may trigger cell death or senescence (74, 75). Our data suggest that the initial response to high Ca^2+^ is the expression of early response genes which is subsequently translated into the expression of genes that enable the tumor cells to strive under the sustained high Ca^2+^ conditions. This is the case of MAGEC2, a cancer-testis antigen that is known to be aberrantly expressed in highly malignant breast and other neoplasms (75). Even though MAGEC2 is now known to play an important role in tumor progression, our study suggests that the up regulation of this gene in malignant TNBC is triggered by high extracellular Ca^2+^ and presumably, breast cancer-induced hypercalcemia in patients with advanced and metastasis-prone disease stages. This is supported by our TCGA data analysis showing an increase in the expression of MAGEC2 in invasive ductal carcinomas compared to normal breast tissues. Although this study established a link between high Ca^2+^ and the expression of MAGEC2, it remains unclear whether the pathway to high Ca^2+^ adaptation inevitably requires extracellular Ca^2+^ sensing/signaling *via* the CaSR and/or Ca^2+^ entry mechanisms including Ca^2+^ activated nonselective cation channels (36). Further studies will be necessary to determine the fate of high Ca^2+^-adapted TNBCs *in vitro* and *in vivo*, and whether high Ca^2+^ inducible expression of malignancy-associated genes is reversible following treatment with Ca^2+^ lowering drugs. Further studies are also warranted to validate MAGEC2 and other high Ca^2+^-inducible genes as biomarkers for hypercalcemia modulated cancer progression and metastasis.

## Conclusion

Data from this study suggests for the first time that the aberrant expression of MAGEC2 and presumably related cancer-testis antigens in malignant solid tumors is triggered at least in part by high circulating Ca^2+^. This study also provides novel mechanistic insights into the hitherto unappreciated oncogenic effects of high extracellular Ca^2+^, especially in advanced breast cancers.

## Data Availability Statement

The original contributions presented in the study are publicly available. This data can be found here: https://www.ncbi.nlm.nih.gov/geo/, GSE189520.

## Author Contributions

This work was conceptualized by AS. The methodology, data generation and analysis was conducted by HB, SP, SEW, SDW, OK, CN, DW, and AS. Initial writing, editing and reviewing of the manuscript was conducted by HB and AS and the final review of the manuscript and editing was conducted by HB, SEW, DW, SDW, OK, CN, JO, and AS. All authors have read and agreed to the published version of the manuscript.

## Funding

This work was supported by the NIH/NIGMS 5SC2 CA170244 and SC1 CA211030 (to AS), 8U54 MD007593, Meharry Translational Research Center (MeTRC) and P50CA098131 (Vanderbilt-Ingram Cancer Center SPORE in Breast Cancer). This study used resources from the Meharry RCMI Infrastructure Core (CRISALIS) which is supported by U54 MD007586. HB was supported by U54 CA163069; SEW, DW, and SDW were supported by R25 GM059994; and CN was supported by the Federal Work Study (FWS) program. The content of this manuscript is solely the responsibility of the authors and does not necessarily represent the official views of the National Institutes of Health.

## Conflict of Interest

The authors declare that the research was conducted in the absence of any commercial or financial relationships that could be construed as a potential conflict of interest.

## Publisher’s Note

All claims expressed in this article are solely those of the authors and do not necessarily represent those of their affiliated organizations, or those of the publisher, the editors and the reviewers. Any product that may be evaluated in this article, or claim that may be made by its manufacturer, is not guaranteed or endorsed by the publisher.
