# Supplementary material for: Identification of MAGEC2/CT10 as a High Calcium-Inducible Gene in Triple-Negative Breast Cancer
Source: Front Endocrinol (Lausanne). 2022 Mar 10;13:816598. doi: 10.3389/fendo.2022.816598 (PMC8959981; doi:10.3389/fendo.2022.816598)
Supplement: Supplementary file 1 [file DataSheet_1.pdf]

# *Supplementary Material*

## 1 Supplementary Tables

**Table S1. TaqMan gene expression assays used in this study**

| Assay ID | Gene symbol | Gene Description |
|---|---|---|
| **Hs00171851_m1** | **FOSB** | FosB proto-oncogene, AP-1 transcription factor subunit |
| **Hs04194186_s1** | **FOS** | Fos proto-oncogene, AP-1 transcription factor subunit |
| **Hs01114274_m1** | **IL24** | Interleukin 24 |
| **Hs01055413_g1** | IL11 | Interleukin 11 |
| **Hs00985641_m1** | IL6 | Interleukin 6 |
| **Hs01854535_s1** | MAGEC2 | MAGE family member C2 |
| **Hs00152928_m1** | EGR1 | Early growth response 1 |
| **Hs01010736_m1** | SERPINB2 | Serpin family B member 2 |
| **Hs00189369_m1** | DNM1 | Dynamin 1 |
| **Hs00236216_m1** | ITGB4 | Integrin subunit beta 4 |
| **Hs00161638_m1** | SCG5 | Secretogranin V |
| **Hs01084940_m1** | NCF2 | neutrophil cytosolic factor 2 |
| **Hs01060665_g1** | ACTB* | Actin beta |
| **Hs02786624_g1** | GAPDH* | Glyceraldehyde-3-phosphate dehydrogenase |

* ACTB and GAPDH were used as housekeeping gene expression controls.

**Table S2. Up regulated genes at high calcium**

| GENE SYMBOL | GENE DESCRIPTION | FOLD CHANGE | P-VALUE |
|---|---|---|---|
| FOSB | FBJ murine osteosarcoma viral oncogene homolog B | 3.1307 | 0.0054 |
| FOS | FBJ murine osteosarcoma viral oncogene homolog | 2.7834 | 0.0051 |
| MAGEC2 | Melanoma antigen family C, 2 | 2.5627 | 0.0039 |
| SERPINB2 | Serpin peptidase inhibitor, clade B (ovalbumin), member 2 | 2.5328 | 0.0005 |
| NCF2 | Neutrophil cytosolic factor 2 | 2.2339 | 0.0010 |
| ID3 | Inhibitor of DNA binding 3, dominant negative helix-loop-helix | 2.1861 | 0.0173 |
| ACTG2 | Actin, gamma 2, smooth muscle, enteric | 2.1772 | 0.0134 |
| SSX1 | Synovial sarcoma, X breakpoint 1 | 2.1701 | 0.0208 |
| PDE1C | Phosphodiesterase 1C, calmodulin-dependent 70kDa | 2.0546 | 0.0206 |
| SPANXE | SPANX family, member E | 2.0388 | 0.0075 |
| SNORA14B | Small nucleolar RNA, H/ACA box 14B | 2.0051 | 0.0030 |
| FRMD3 | FERM domain containing 3 | 1.9955 | 0.0016 |
| FRMPD4 | FERM and PDZ domain containing 4 | 1.9895 | 0.0100 |
| IL24 | Interleukin 24 | 1.9708 | 0.0019 |
| IL11 | Interleukin 11 | 1.9645 | 0.0003 |
| SCG5 | Secretogranin V (7B2 protein) | 1.9512 | 0.0118 |
| SNORA1 | Small nucleolar RNA, H/ACA box 1 | 1.9325 | 0.0004 |
| ZNF257 | Zinc finger protein 257 | 1.8978 | 0.0003 |
| IL6 | Interleukin 6 (interferon, beta 2) | 1.8500 | 0.0447 |
| DCAF8L1 | DDB1 and CUL4 associated factor 8-like 1 | 1.8329 | 0.0229 |
| PLEKHA7 | Pleckstrin homology domain containing, family A member | 1.8110 | 0.0047 |
| EHF | Ets homologous factor | 1.8079 | 0.0119 |
| SPOCK1 | Sparc/osteonectin, cwcv and kazal-like domains proteoglycan (testis) | 1.8060 | 0.0003 |
| ACTR3C | RP3 actin-related protein 3 homolog C (yeast) | 1.8054 | 0.0131 |
| CRLF2 | Cytokine receptor-like factor 2 | 1.7910 | 0.0037 |
| EGR1 | Early growth response 1 | 1.7818 | 0.0501 |
| GJA5 | Gap junction protein, alpha 5, 40kDa | 1.7779 | 0.0175 |
| PSG4 | Pregnancy specific beta-1-glycoprotein 4 | 1.7765 | 0.0392 |
| COL6A3 | Collagen, type VI, alpha 3 | 1.7606 | 0.0003 |

**Table S3. Down regulated genes at high calcium**

| GENE SYMBOL | GENE DESCRIPTION | FOLD CHANGE | P-VALUE |
|---|---|---|---|
| C15orf51 | Dynamin 1 pseudogene | -4.13153 | 0.00034 |
| REXO1L1 | RNA exonuclease 1 homolog (S. cerevisiae)-like 1 | -2.2933 | 0.00670 |
| NRK | Nik related kinase | -2.19675 | 0.01087 |
| REXO1L2P | RNA exonuclease 1 homolog (S. cerevisiae)-like 2 (pseudo | -2.15397 | 0.00065 |
| HLA-DRA | Major histocompatibility complex, class II, DR alpha | -2.13535 | 0.00013 |
| EPB41L3 | Erythrocyte membrane protein band 4.1-like 3 | -2.08737 | 0.02883 |
| RFPL4A | Ret finger protein-like 4A | -2.03611 | 0.00189 |
| LCE2D | Late cornified envelope 2D | -2.03231 | 0.00019 |
| FOLH1 | Folate hydrolase (prostate-specific membrane antigen) 1 | -1.88823 | 0.01010 |
| DUX4L4 | Double homeobox 4 like 4 | -1.87542 | 0.03024 |
| LOC349196 | Hypothetical LOC349196 | -1.84076 | 0.00020 |
| LY86-AS | LY86 antisense RNA (non-protein coding) | -1.83354 | 0.00210 |
| ICOSLG | Inducible T-cell co-stimulator ligand | -1.83162 | 0.00433 |
| ABCA1 | ATP-binding cassette, sub-family A (ABC1), member 1 | -1.821 | 0.00909 |
| TNFSF18 | Tumor necrosis factor (ligand) superfamily, member 18 | -1.81363 | 0.00013 |
| HLA-DRB3 | Major histocompatibility complex, class II, DR beta 3 | -1.81248 | 0.00030 |
| LOC10013247 | Hypothetical LOC100132147 | -1.79995 | 0.01067 |
| MUC12 | Mucin 12, cell surface associated | -1.76713 | 0.00076 |
| HLA-DPB1 | Major histocompatibility complex, class II, DP beta 1 | -1.74847 | 0.00033 |
| IGHA1 | Immunoglobulin heavy constant alpha 1 | -1.73004 | 0.00147 |
| ZFPM2 | Zinc finger protein, multitype 2 | -1.71818 | 0.01157 |
| SCD | Stearoyl-CoA desaturase (delta-9-desaturase) | -1.71338 | 0.08876 |
| ASB2 | Ankyrin repeat and SOCS box-containing 2 | -1.71158 | 0.00926 |
| IGHD | Immunoglobulin heavy constant delta | -1.70471 | 0.05360 |
| SPRY1 | Sprouty homolog 1, antagonist of FGF signaling (Drosophila) | -1.69598 | 0.00043 |
| HLA-DRB4 | Major histocompatibility complex, class II, DR beta 4 | -1.68976 | 0.00049 |
| HLA-DRB3 | Major histocompatibility complex, class II, DR beta 3 | -1.67505 | 0.00041 |
| GMFG | Glia maturation factor, gamma | -1.66292 | 0.00452 |
| CCNA1 | Cyclin A1 | -1.6413 | 0.00670 |

**Table S4. Pathways modulated by high extracellular calcium**

| PATHWAY | PATHWAY ENTITIES | MATCHED ENTITIES | P-VALUE |
|---|---|---|---|
| Hs Senescence and Autophagy | 106 | 5 | 1.69E-04 |
| Hs Cytokines and Inflammatory Response | 30 | 3 | 1.83E-04 |
| Hs Adipogenesis | 131 | 5 | 4.34E-04 |
| Hs Dissolution of Fibrin Clot | 8 | 2 | 6.41E-04 |
| Hs Vitamin B12 Metabolism | 54 | 3 | 0.001954 |
| Hs TGF Beta Signaling Pathway | 55 | 3 | 0.002428 |
| Hs SREBF and miR33 in cholesterol and lipid homeostasis | 18 | 2 | 0.003026 |
| Hs Folate Metabolism | 68 | 3 | 0.004074 |
| Hs Glutathione metabolism | 20 | 2 | 0.004187 |
| Hs Blood Clotting Cascade | 22 | 2 | 0.005059 |
| Hs Selenium Pathway | 85 | 3 | 0.007709 |
| Hs Epigenetic Stress Regulation | 1 | 1 | 0.009682 |
| Hs MAPK targets-Nuclear events mediated by MAP kinases | 8 | 1 | 0.038172 |
| Hs Myometrial Relaxation and Contraction Pathways | 156 | 3 | 0.039814 |
| Hs Sudden Infant Death Syndrome (SIDS) Susceptibility Pathways | 66 | 2 | 0.040927 |
| Hs Insulin Signaling | 160 | 3 | 0.043075 |

2. **Supplementary Figures**

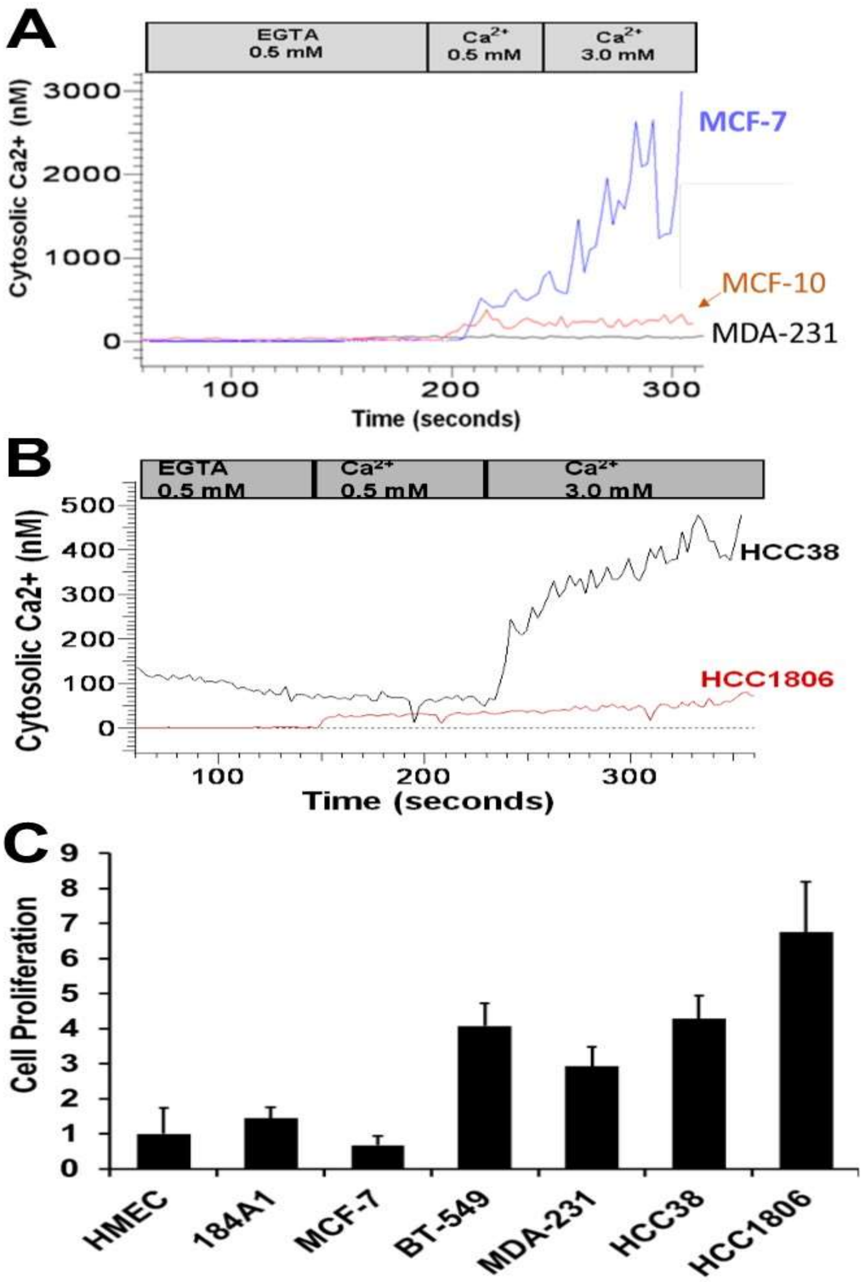


**Figure S1. Differential calcium influx dynamics and survival of Breast epithelial and breast cancer cells at high Ca2+. (A, B)**The indicated cell lines were grown to 80% confluency, harvested by trypsinization and loaded with fura-2 AM for 1 h. Cells were washed twice in $Ca^{2+}$ and $Mg^{2+}$-free Hanks balanced salt solution (HBSS) and intracellular $Ca^{2+}$ levels were measured using the Hitachi F2500 spectrofluorimeter with addition of the indicated solutions. **C)**. Cells were cultured in complete medium supplemented with 5.0 mM Ca2+ and the viability/proliferation was measured as described in Fig. 1E and normalized relative to the primary human epithelial cells (HMEC).

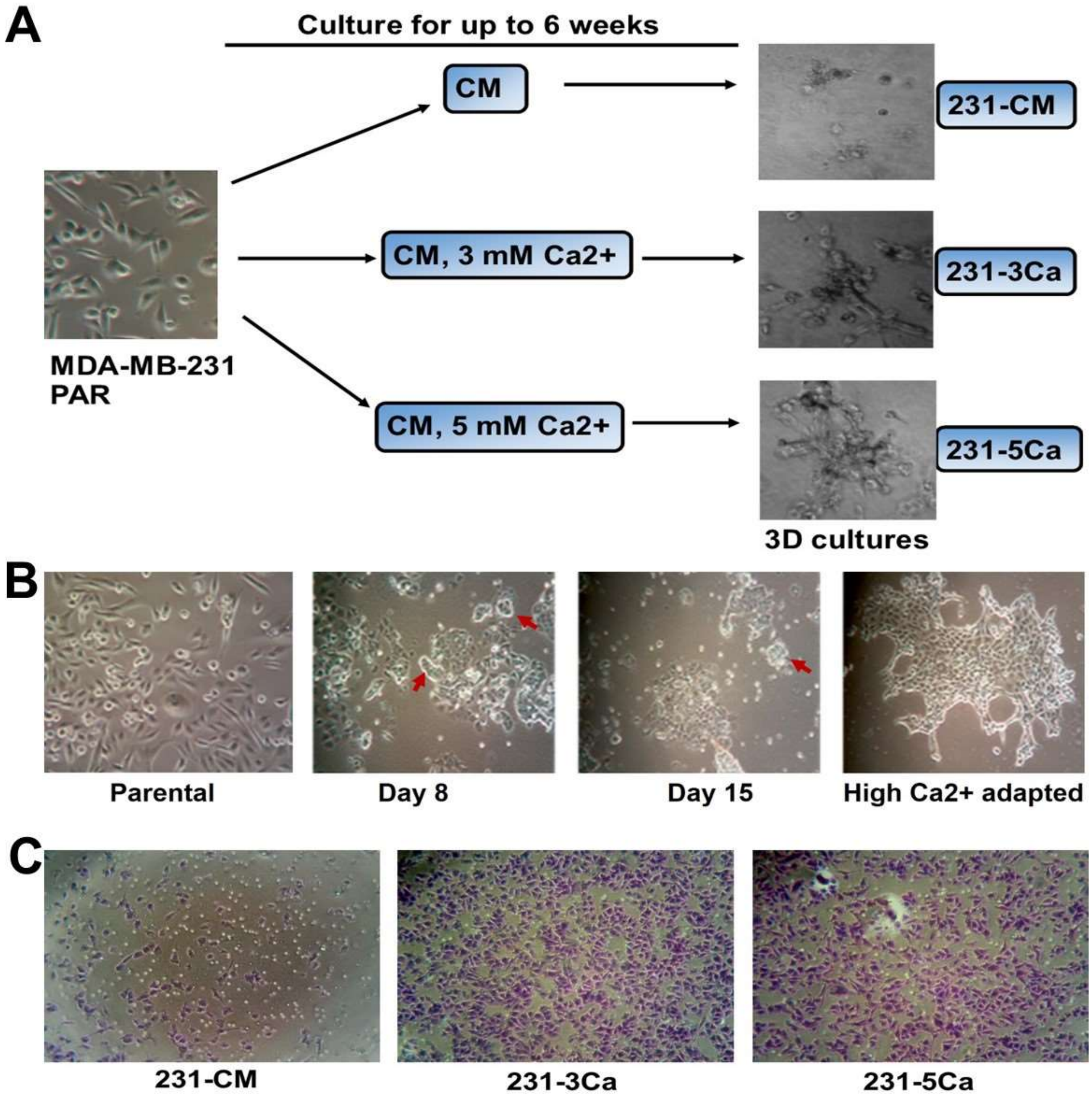


**Figure S2. Adaptation of TNBC cells to high Ca2+ in vitro. A)** Parental MDA-MB-231 cells were cultured in complete medium or complete medium supplemented with 3.0 or 5.0 mM Ca2+ for up to 6 weeks. **B**) The morphology of the cells during the continuous culture at high Ca2+. Arrows indicate colonies of surviving cells by day 8 of the selection process. C) Migration of control and high Ca2+ adapted MDA-MB-231 cells. Cells were plated in Boyden chambers in serum-free medium and complete medium containing 10% FBS was used as chemoattractant. Shown are representative images from at least three fields.

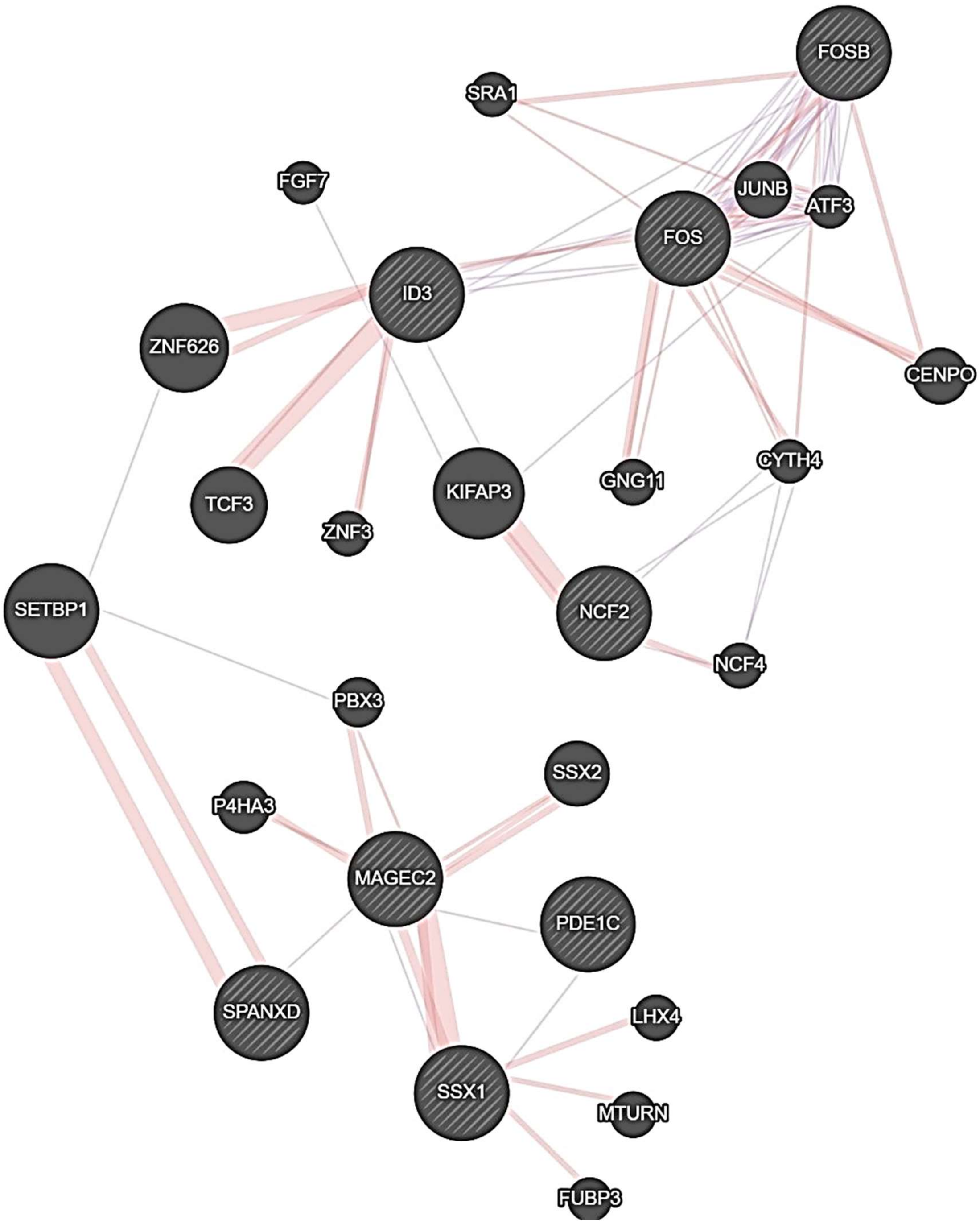


**Figure S3. Biological network relationship of up regulated (high $Ca^{2+}$ inducible) genes.** The hashed nodes indicate genes identified to be up regulated at high Ca2+ in MDA-MB-231 TNBC cells and the physical interactions co-expression analyzed by using genemania (https://genemania.org/) are indicated by pink and purple connections respectively.

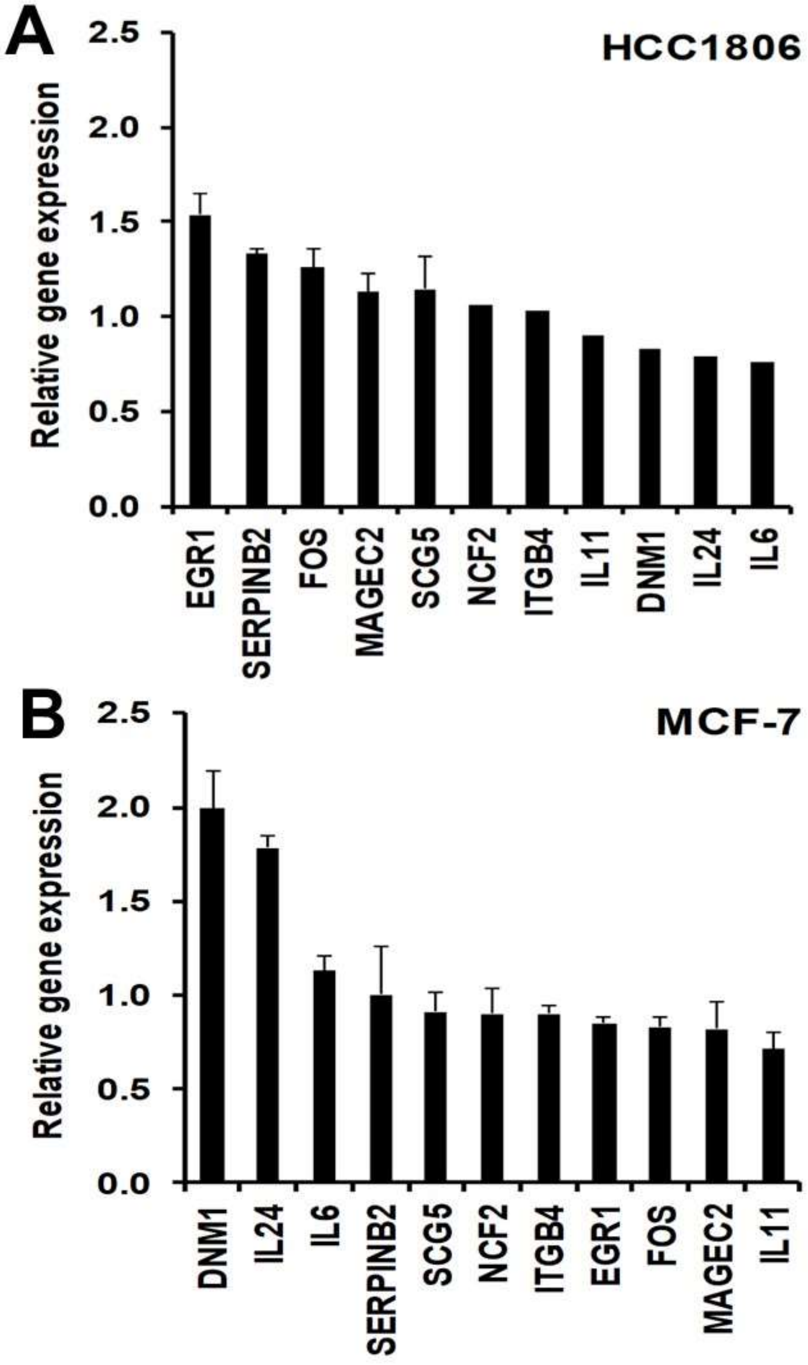


**Figure S4. Validation of upregulated genes in high Ca2+ treated breast cancer cells**. The indicated high Ca2+ inducible genes were validated by RT-PCR in HCC1806 (A) and MCF-7 (B) breast cancer cells. Bars represent gene expression relative to GAPDH control.

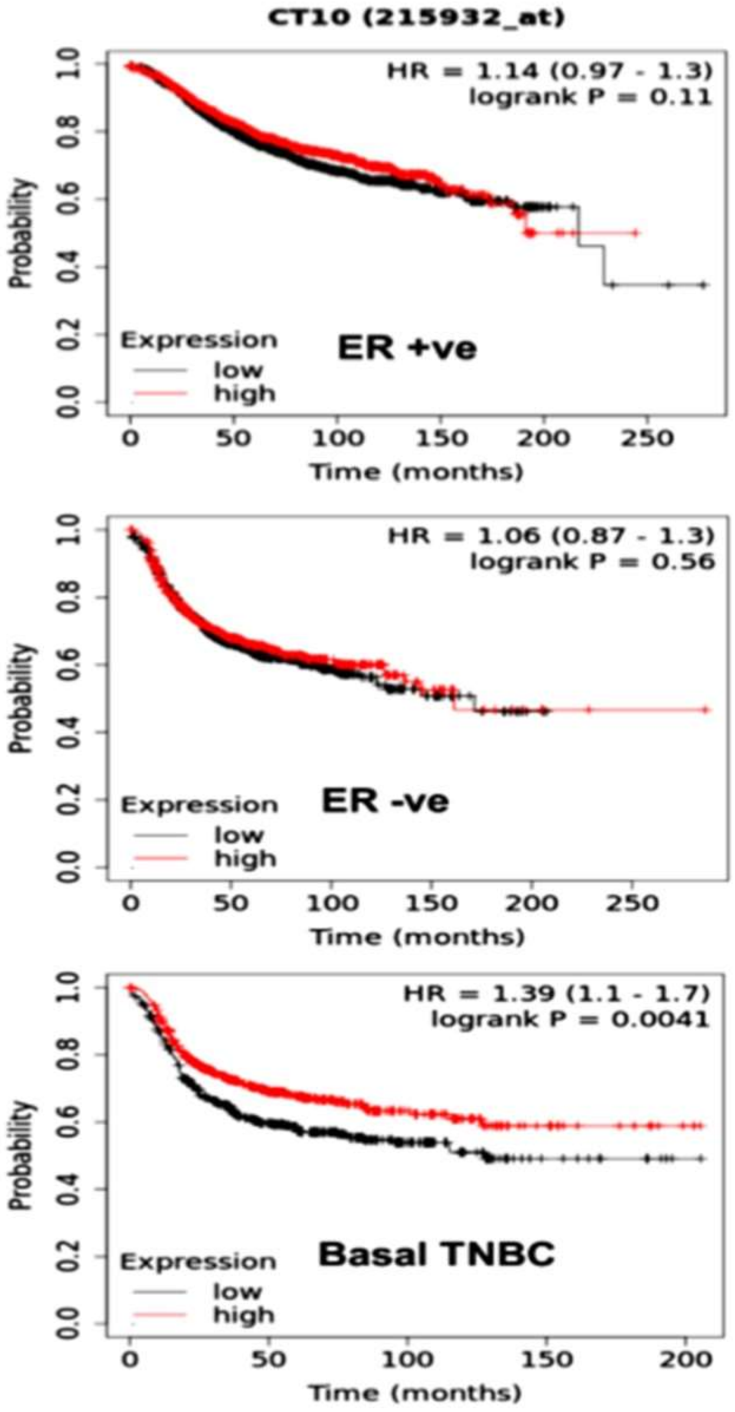


**Figure S5. Clinical relevance of MAGEC2 expression in basal-like breast cancer relative to ER positive and ER negative breast cancer**. The relapse-free survival of patients with ER-positive and ER-negative breast cancer as well as basal-like TNBC was analyzed as a function of high (red) and low (black) expression levels above and below the median value respectively, by using the Kaplan Meier survival analysis tool, KM plotter for breast cancer.
